# Treatment of 5-fluorouracil refractory metastatic colorectal cancer: an Australian population-based analysis

**DOI:** 10.1038/sj.bjc.6603590

**Published:** 2007-02-06

**Authors:** D Damianovich, M Adena, N C Tebbutt

**Affiliations:** 1 Ludwig Institute for Cancer Research, Austin Health, Melbourne, Victoria, Australia; 2 Covance Pty Ltd, Canberra, Australian Capital Territory, Australia

**Keywords:** metastatic colorectal cancer, irinotecan, oxaliplatin

## Abstract

Randomised trials have established the importance of oxaliplatin (O) and irinotecan (I) in advanced colorectal cancer (CRC). However, patients enrolled in clinical studies represent a restricted population and little is known about the use of O and I in the general population and the subsequent outcomes outside clinical studies. We used the Australian Health Insurance Commission (HIC) database to describe prescribing patterns of O and I and their impact on survival in all patients with 5-fluorouracil (5-FU) refractory CRC in Australia in 2002 and 2003. In 2999 patients, there was a marked increase in initial treatment with O rather than I; 48% of patients received O first in 2002 *vs* 66% in 2003 (*P*<0.001). Overall 40–45% of patients received both O and I; however, younger patients were more likely to receive both drugs (*P*<0.001). After 5-FU failure and treatment with O or I, the proportion of patients surviving 6 or 12 months was estimated to be 0.67 (95% CI, 0.66–0.69) and 0.42 (95% CI, 0.40–0.44), respectively. Survival was superior for patients who received both O and I; however, the sequence of agents had no impact. Older patients (⩾70 years) had inferior survival no matter which drug was used as initial treatment. Analysis of the Australian HIC database provides a valuable means of assessing patterns of use and outcomes of new therapies.

Colorectal cancer (CRC) is the second most common cause of cancer death in Australia. Approximately 40% of diagnosed patients will ultimately die of the disease ((Australian Institute of Health and Welfare (AIHW) and Australian Association of Cancer Registries (AACR), 2004)).

Fluorouracil (5-FU) and its modulator leucovorin (LV) have been used for the treatment of metastatic CRC for years. Irinotecan (I), an inhibitor of topoisomerase I, was one of the first cytostatics to show a survival benefit in the treatment of 5-FU-resistant disease (Cunningham *et al*, 1998; Rougier *et al*, 1998). This was confirmed in the first-line setting when irinotecan was combined with either bolus (Saltz *et al*, 2000) or infusional (Douillard *et al*, 2000) 5-FU. A similar progression-free survival benefit was achieved with the combination of the platinum analogue, oxaliplatin (O), with 5-FU (De Gramont *et al*, 2000; Grothey *et al*, 2002).

However, Grothey *et al* (2004) recently showed the importance of making all active agents, 5-FU/LV, oxaliplatin and irinotecan, available to patients during the course of their disease to achieve optimal survival.

Although the benefit of I and O in the treatment of metastatic CRC has been clearly established in clinical trials, few studies have evaluated the use of these agents in the general population outside clinical trials.

The Health Insurance Commission (HIC), now Medicare Australia, is a Government organisation whose main task is to provide to all Australian residents equity of medical care under the state insurance scheme, Medicare, and to make a range of necessary prescription medicines available at affordable prices through the Pharmaceutical Benefit Scheme (PBS). This scheme provides access to approved drugs for both public and private patients as private health funds do not provide drug reimbursement in Australia.

Given the expense of I and O and the funding arrangements within the health system of Australia, almost all use of these drugs by metastatic CRC patients in Australia is subsidised under the PBS and thus recorded in the database. The PBS item numbers for I and O are specific for metastatic CRC as they are not PBS approved for other indications.

In this study, we have used the HIC database to describe trends in prescribing patterns of oxaliplatin and irinotecan and to evaluate survival outcomes analysed by treatment sequence and patient demographics.

This analysis provides information about patterns of use of O and I and estimates of survival for the general population of Australian patients, rather than being restricted to the population enrolled in clinical studies.

This means that these results may be of interest both within and outside Australia, particularly as we are not aware of any reports of comparable data for other countries.

## PATIENTS AND METHODS

The research undertaken in this analysis was approved by Austin Health Human Research Ethics Committee. The HIC database was searched to identify the patients with metastatic CRC who had one or more scripts for I or O supplied under the PBS between 1 January 2001 and 31 December 2004. For the period of this analysis, I and O were approved under the PBS only for patients with metastatic CRC who had failed 5-FU and LV. Thus, during this time interval patients would generally receive PBS approval for O or I as second- or third-line treatment of metastatic disease after 5FU failure. The cohort of patients followed was defined as those patients who received their first supply (no supply of either agent previously) of I or O between 1 January 2002 and 31 December 2003.

The following data were available for each patient: sex, age at 1 January 2002, date of first supply of I or O, state/territory of supplier, region (city/country) of supplier and date of last supply of any PBS item. The HIC summarised the patient-level data into tables, which we analysed.

The proportion of patients whose first supply was I or O as well as the proportion of patients switching from O to I and vice versa was analysed using logistic regression to determine how this proportion varied with year, sex, age group and location.

Exact date of death was not routinely recorded on the HIC database. However, terminally ill patients are expected to require regular PBS-approved medications such as nonsteroidal analgesics, morphine derivates, sedatives, laxatives as well as medications for other comorbid conditions. Ongoing supply of any PBS item indicates ongoing survival of an individual patient, whereas cessation of supply of PBS items was used in the analysis as a surrogate indicator of death. Six- and 12-month survival was estimated by calculating the proportion of patients with recorded PBS prescriptions of any type at least 6 and 12 months after the date of their first supply of either I or O.

The number of patients still alive at 6 and 12 months, as a proportion of the number of patients who started a particular treatment for metastatic CRC, was analysed using logistic regression.

We have verified the validity of this surrogate marker of death using a data set of 548 patients for whom the actual date of death had been recorded. The median difference between the last date of PBS supply and the actual recorded date of death was 7 days. For patients with a recorded date of death, the estimated proportion of patients still receiving PBS supplies (of any drug) 6 or 12 months after the supply of I or O was within 2 percentage points of the corresponding estimate derived from the actual recorded date of death.

## RESULTS

### Patient demographics

By searching the Australian HIC database, 1465 new patients with 5FU-refractory metastatic CRC starting either I- or O-based chemotherapy were found in 2002 and 1534 in the year 2003. These patients had no supply of I or O in 2001 and are therefore likely to have been naïve to these agents. This is estimated to represent approximately 90% of the population receiving these agents as patients receiving these agents through the Repatriation PBS (retired servicemen or women) (4% of prescriptions) and patients receiving these agents in clinical trials would not be identified with this analysis.

The proportion of male and female patients, the overall age distribution and the age distribution for male and female patients were similar in 2002 and 2003. Approximately 23% of identified patients were 70 years and older and 2% were 80 years and older.

### Which treatment is used first?

**Figure 1:**
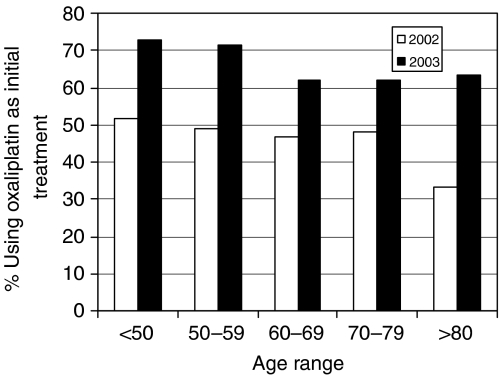
New I or O patients first supplied with O, by age group and year (^*^P<0.001; 2003 *vs* 2002 and young *vs* old).

There was a marked change to an earlier use of O in 5-FU pretreated patients in 2003, as the overall percentage of patients who were treated with O first was greater in 2003 than 2002 (66 *vs* 48%, *P*<0.001). The differences between 2002 and 2003 were greater for younger than for older patients (Figure 1); however, it was evident across all age groups. The change in the pattern of use of I and O was observed consistently across all states in Australia.

### How many patients started on I switch to O?

The overall percentage of patients switching from I to O was the same for both years (45%). However, this percentage was greater for younger than for older patients (*P*<0.001) (Table 1a), which could be because more intensive treatments are usually prescribed to younger and fitter patients. A similar pattern was observed in both 2002 and 2003 and for male and female patients.

### How many patients started on O switched to I?

Of the 697 patients who started O in 2002, 40% switched to I. This percentage was the same in 2003. Similar to the previous observation with I, this percentage was greater for younger than for older patients (Table 1b).

### Survival of patients

Of the 2999 patients who had their first supply of either I or O in 2002 or 2003, 2024 were known to be still alive 6 months after the first supply of their respective treatment. The proportion of patients surviving at 6 months was therefore estimated to be 0.67 (Table 2a).

At 12 months, 1262 were still alive with the estimated proportion of patients surviving at 12 months being 0.42 (95% CI, 0.40–0.44). The patients first supplied with O had 6- and 12-month survival, 4 percentage points higher than the patients first supplied with I (each *P*<0.05; Table 2a). This difference is statistically significant, but may reflect differences in the baseline characteristics of the patients.

The largest difference in survival was observed between the patients who received both O and I and those who received one treatment only (*P*<0.001; Table 2b). However, for patients who received both O and I, there was no difference in survival according to treatment sequence (I → O *vs* O → I). The greater survival in patients receiving both drugs sequentially might reflect the fact that patients who have better prognosis disease or better performance status are able to receive two or sometimes three lines of treatment as opposed to patients with worse disease prognosis.

In contrast to these results, survival estimates from the time of initiation of the last treatment were very similar, irrespective of the agent and whether one or both agents had been used. Thus, when the last treatment was O, 6- and 12-month survival proportions were 0.55 and 0.30 for patients who received only O and were 0.55 and 0.25 for patients who had received prior I. Overall, the proportions surviving 6 months was 0.46 when the last treatment received was I only and 0.5 when the last treatment was I preceded by O. The corresponding 12-month survival proportions were 0.25 and 0.22, respectively.

Thus, patients have a similar chance of survival after the last treatment with either O or I, irrespective of prior treatment sequence or number of prior agents.

### Age and sex

Six- and 12-month survival after initial or treatment only with either I or O varies with sex and age group. Higher survival was observed in younger patients irrespective of the type of treatment. Higher survival for younger male than female patients was observed when the initial treatment was I. There was less difference in survival between male and female patients when the initial treatment was O.

## DISCUSSION

The value of I or O in the treatment of either 5-FU-pretreated or chemotherapy-naïve patients with metastatic CRC has been well established in randomised clinical trials. Both drugs showed near-identical response rates, progression-free and overall survival times when they were combined with infusional 5-FU and when directly compared (Tournigand *et al*, 2004; Colucci *et al*, 2005). Staged sequencing of I and O-based regimens can achieve median survival times of approximately 20 months in patients with metastatic CRC (Tournigand *et al*, 2004).

However, patients in clinical trials represent a restricted population. They are usually younger, fitter, and without comorbidities, which could potentially influence the outcomes. There are no published data on the use of modern treatments in metastatic CRC in the general population.

This study analyses patterns of use of I and O in Australia over a period of 2 years. During that time, both drugs were approved only for metastatic CRC patients who had failed initial 5-FU-based treatment.

Searching the Australian HIC database, 2999 patients with 5-FU refractory metastatic CRC were found to receive initial treatment with either I- or O-based regimen in the years 2002 and 2003, which is roughly two-thirds of all patients expected to be treated for metastatic disease. The remaining one-third of patients was likely to receive either single-agent 5-FU or palliative care only. These estimates are based on the observations that approximately 15–20% of all patients diagnosed with CRC receive palliative chemotherapy for their metastatic disease each year in Australia (Spigelman and McGrath, 2000; McLeish *et al*, 2002). That is about 1900–2600 patients, taking into account 12 900 patients newly diagnosed with CRC in the year 2001 ((Australian Institute of Health and Welfare (AIHW) and Australian Association of Cancer Registries (AACR), 2004)).

### Demographic trends

The distribution by age and sex was similar for both years. Although patients aged ⩾70 years account for 55% of newly diagnosed CRC cases in Australia ((Australian Institute of Health and Welfare (AIHW) and Australian Association of Cancer Registries (AACR), 2004)), in our cohort only 23% of patients receiving treatment were age 70 or older. However, the proportion of older patients receiving treatment with I or O off-study is greater than that typically observed in clinical trials in either adjuvant (Sargent *et al*, 2001) or metastatic settings (Folprecht *et al*, 2004). This is largely owing to the lack of appropriate trials, a higher number of comorbidities, study-imposed restrictions, and attitudes of physicians, although clinical data demonstrate that age alone is not a sufficient reason to withhold treatment (Aapro *et al*, 2005).

### Patterns of use of I and O

After failing 5-FU, 48% of patients in our cohort received an O-based regimen first in the year 2002 and 66% in 2003. This is probably a reflection of an increased trend for earlier use of O over I in 5-FU-pretreated metastatic CRC after the results of N9741, the Northern Central Cancer Treatment Group trial, had been reported.

In this phase III trial, the overall survival and toxicity profile favoured first-line O (FOLFOX4) over the I combination (IFL). These results may have been due to the fact that 60% of patients in the FOLFOX4 arm received I on progression compared with only 24% receiving second-line O in the IFL arm. In addition, there was also a possibility that the infusional 5-FU in the FOLFOX4 arm may have been more effective than the bolus 5-FU in the IFL regimen (Goldberg *et al*, 2004). In spite of those reservations, O-based regimens have been adopted as the preferred first-line regimen for the treatment of metastatic CRC in the USA. It seems likely that this study has also influenced Australian practice. Interestingly, the trend to use O earlier was observed consistently across all states in Australia.

Grothey *et al* (2004), in a pooled analysis of recently published phase III trials, highlighted the importance of sequencing of 5-FU, I, and O in the treatment of metastatic CRC. They found a strong correlation between reported median survival and the percentage of patients who received all three available drugs.

In our 5-FU-pretreated population, the overall percentage of patients switching from an I- to an O-based regimen was 45% in both 2002 and 2003. In both years, 40% of patients receiving second-line O subsequently switched to third-line I.

These switch rates are comparable with those observed in the recently reported American N9841 phase III trial with a similar study population to ours. In this study, patients who previously failed 5-FU were first randomised to receive either 3-weekly I or 2-weekly FOLFOX4 then crossed over to the other regimen at disease progression. Fifty-one percent of patients receiving second-line I switched to third-line FOLFOX4 and 38% of patients starting FOLFOX4 subsequently received third-line I (Rowland *et al*, 2005).

Irrespective of the treatment sequence, in our study, the switch rates were higher for younger than for older patients (*P*<0.001), which could be the result of a better performance status of the younger population or a tendency to treat older patients less intensively.

### Survival outcomes with different types of treatment and treatment sequences

In the Australian population, estimated 6- and 12-month survival after 5FU failure was 66 and 40% in 2002 and 69 and 44% in 2003, respectively. The differences in survival between 2002 and 2003 were not statistically significant. When survival was analysed by the first treatment supplied, patients who received O first appeared to have both better 6-month (69 *vs* 65%, *P*<0.05) and 12-month survival (44 *vs* 40%, *P*<0.05) compared with patients receiving I first after 5-FU failure. As this is a non-randomised comparison, these differences in survival observed in our study may reflect different patient baseline characteristics.

Six and 12-month survival rates for second-line O or I in our study were slightly lower but similar to the ones reported in clinical trials (Table 3), which probably reflects the fact that the patients enrolled in clinical trials are usually younger and fitter with fewer comorbidities than the ones treated outside clinical trials. Thus, the use of second-line I after 5-FU failure in clinical studies was associated with 6- and 12-month survival rates ranging from 70 to 80% and 41 to 57%. The corresponding 6- and 12-month survival rates after second-line O ranged from 82 to 83% and 57 to 60% (Table 3).

In accordance with Grothey's analysis (Grothey *et al*, 2004), those 5-FU refractory patients who received both I and O during the course of their treatment, irrespective of the treatment sequence, had the longest 6- and 12-month survivals (89–91 and 58–64%, respectively). Although likely to be at least partly explained by a treatment effect, this result is confounded by the fact that patients with less aggressive disease are able to live long enough to be able to receive all available agents.

### Influence of age and sex on survival

We found higher survival for younger patients irrespective of the type of initial treatment, perhaps because clinicians treat older patients less intensively.

This is contrary to the observations from randomised clinical trials, which showed similar efficacy and toxicity rates for 5-FU, I or O in selected older patients (⩾70 years) compared with younger patients (Botto, 2004; Folprecht *et al*, 2004; Stewart *et al*, 2004; Goldberg *et al*, 2006).

### CONCLUSIONS

In conclusion, this study shows the feasibility of the analysis of the HIC database for evaluation of prescribing trends and treatment outcomes in real practice.

As anticipated, the survival rates observed in the general population after the use of I and O are not as high as those observed in prospective randomised clinical studies. Nevertheless, these data provide important information about realistic outcomes that may be achieved with these agents.

It is reassuring that many outcomes from this study are similar to observations from prospective clinical trials, indicating that these results are generalisable. For instance, our study shows the importance of making all three active agents (5-FU, I and O) available to metastatic CRC patients in order to achieve maximum benefit. Similarly, there was no clear indication that any particular sequence of agents is preferable.

The general population also includes older patients and patients with comorbidities who are frequently excluded from clinical trials. In spite of the availability of these drugs to all patients, the older population in real life is less likely to be treated with both I and O. Contrary to observations from clinical trials with highly selected older patients, their survival outcomes appear worse compared with younger age groups. This is likely to be due to generally worse performance status and a larger number of comorbid conditions.

Newer biological agents including bevacizumab and cetuximab have now shown benefits in clinical trials in advanced CRC. These agents have not yet received PBS approval in Australia. However, if they were to receive approval in the future, we believe that a similar analysis to this would be valuable to define the benefits associated with these agents in the general population.

## Figures and Tables

**Table 1a:** New I patients switching to O, by sex, age group and year of starting I (percent and overall proportion)

	**New patients in 2002**		**New patients in 2003**	
**Age group**	**Male (%)**	**Female (%)**	**Male (%)**	**Female (%)**
<50	58	55	55	48
59–59	52	53	52	58
60–69	43	35%	45	45
70–79	44	33	38	26
⩾80	27	29	17	100
Overall	45 (344/768)		45 (234/525)	

I, irinotecan; O, oxaliplatin.

**Table 1b:** New O patients switching to I, by sex, age group and year of starting O (percent and overall proportion)

	**New patients in 2002**		**New patients in 2003**	
**Age group**	**Male (%)**	**Female (%**	**Male (%)**	**Female (%)**
<50	46	46	53	55
50–59	45	37	43	41
60–69	45	36	42	37
70–79	34	30	29	24
⩾80	25	0	100	18
Overall	40 (276/697)		40 (401/1 009)	

I, irinotecan; O, oxaliplatin.

**Table 2a:** Proportion of patients alive at 6 and 12 months after receiving I or O as initial treatment

	**Either I or O**	**First supply I**	**First supply O**
Proportion alive at 6 months	0.67	0.65	0.69
Proportion alive at 12 months	0.42	0.40	0.44

I, irinotecan; O, oxaliplatin.

**Table 2b:** Proportion of patients alive at 6 and 12 months after receiving either I or O alone or both agents as sequenced treatments

	**I only**	**O only**	**I then O**	**O then I**
Proportion alive at 6 months	0.46	0.55	0.89	0.91
Proportion alive at 12 months	0.25	0.30	0.58	0.61

I, irinotecan; O, oxaliplatin.

**Table 3:** Survival of 5-FU refractory pts treated with either O or I in clinical trials

	**Overall survival**						
	**6-month (%)**		**12-month (%)**		**Median (months)**		
**Study**	**I**	**O**	**I**	**O**	**I**	**O**	***P***-**value**
Second-line							
(Fuchs *et al*, 2003)	70	—	41	—	9.9	—	
(Maughan, 2005)	80	83	57	60	14.8	15.2	NS
(Haller *et al*, 2004)	73	82 (I+O)	48	57 (I+O)	11.1	13.4 (I+O)	0.0072
							
Third-line							
(Rowland *et al*, 2005)	71	75	35	39	8.7	10	NS

I, irinotecan; NS: nonsignificant; O, oxaliplatin.
